# Abiotic Determinants of the Historical Buildings Biodeterioration in the Former Auschwitz II – Birkenau Concentration and Extermination Camp

**DOI:** 10.1371/journal.pone.0109402

**Published:** 2014-10-03

**Authors:** Małgorzata Piotrowska, Anna Otlewska, Katarzyna Rajkowska, Anna Koziróg, Mariusz Hachułka, Paulina Nowicka-Krawczyk, Grzegorz J. Wolski, Beata Gutarowska, Alina Kunicka-Styczyńska, Agnieszka Żydzik-Białek

**Affiliations:** 1 Lodz University of Technology, Institute of Fermentation Technology and Microbiology, Lodz, Poland; 2 University of Lodz, Department of Algology and Mycology, Lodz, Poland; 3 University of Lodz, Department of Geobotany and Plant Ecology, Lodz, Poland; 4 Auschwitz–Birkenau State Museum, Oświęcim, Poland; University of Akron, United States of America

## Abstract

The paper presents the results of a study conducted at the Auschwitz-Birkenau State Museum in Oświęcim on the occurrence of biodeterioration. Visual assessment of the buildings revealed signs of deterioration of the buildings in the form of dampness, bulging and crumbling plaster, and wood fiber splitting. The external surfaces, and especially the concrete strips and ground immediately adjoining the buildings, were colonized by bryophytes, lichens, and algae. These organisms developed most intensively close to the ground on the northern sides of the buildings. Inside the buildings, molds and bacteria were not found to develop actively, while algae and wood-decaying fungi occurred locally. The factors conducive to biological corrosion in the studied buildings were excessive dampness of structural partitions close to the ground and a relative air humidity of above 70%, which was connected to ineffective moisture insulation. The influence of temperature was smaller, as it mostly affected the quantitative composition of the microorganisms and the qualitative composition of the algae. Also the impact of light was not very strong, but it was conducive to algae growth.

## Introduction

Historical buildings, exposed to the action of atmospheric conditions, are subjected to varying humidity, temperature, and lighting levels, as well as to chemical contamination [1], [2]. Among the microorganisms responsible for biological corrosion, bacteria and fungi tolerate low temperature and humidity, as well as a wide range of pH levels, and they also easily adjust to unfavorable environmental conditions. The presence of some organic matter, such as dust, is sufficient for them to grow. In turn, the development and metabolic activity of algae, lichens, and bryophytes depend on the presence of carbon dioxide, lighting, and an adequate amount of water [3], [4], [5].

The composition of the biocenosis of the buildings is influenced by many abiotic factors linked to the materials of which the structural elements and equipment are made, as well as connected to the environment in which the buildings are situated. As the presence of water in the environment is a condition necessary for the development of all living organisms, of critical importance to the process of deterioration is long-term excessive relative humidity of the air and dampness of building materials. The latter parameter is closely linked to the technical condition of the buildings. Dampness may result from technical causes (faulty structure of the building, inadequate thermal insulation of structural partitions, absent or ineffective moisture insulation, a leaking roof, etc.) or fortuitous events (e.g., a flood) [5], [6], [7]. The colonization of buildings by micro- and macroorganisms leads to biodeterioration, which is defined as undesirable physical, chemical, and mechanical changes in buildings. The direction and intensity of biodeterioration is determined by the chemical composition of the materials, their porosity, water permeability, and the availability of nutrients [8]. Inorganic materials, such as bricks or plaster, undergo corrosion caused by biological factors as the metabolites of microorganisms (usually organic acids) react with the components of the materials. The production of organic acids leads to increased solubility of some components of stones as a result of the formation of salts or chelates with metal cations (calcium, aluminum, silicon, and iron). Such compounds may be washed out from the surface, resulting in reduced compressive strength [9], [10]. Organic materials, such as wood, are biodegraded by the action of the hydrolytic enzymes of the colonizing organisms as a result of decomposition of cellulose, hemicellulose, and lignins [2], [11].

The macroscopic symptoms of biodeterioration include visible surface defects, such as altered structure, fiber splitting, pitting, microbial growths, discoloration, bulging plaster, and peeling paint. An effect that is dangerous to the durability of buildings is change of their mechanical properties, including strength, which is particularly important in the case of structural elements [8].

The former concentration and extermination camps Auschwitz I and Auschwitz II-Birkenau, which are now part of the Auschwitz-Birkenau State Museum, comprise many historical buildings which constitute a memorial for the tragic events of World War II and are a symbol of the Holocaust and Nazi crimes. The area of the former camp Auschwitz-Birkenau is 191 ha. The museum is responsible for the conservation of, among others, 155 buildings, approximately 300 relics, 35 watchtowers, 4 ruined gas chambers and crematoria, 2 water treatment plants, shelters, as well as many kilometers of railroad tracks, roads, irrigation/drainage ditches, sewers, fences, and gates. To date, 45 brick barracks on section BI and 19 wooden barracks on section BIIa former camp Auschwitz II - Birkenau, have been preserved. Due to the climatic conditions, that is, variable temperature, relative air humidity, and dampness of surfaces inside the buildings, as well as the absence of heating or efficient moisture insulation, these buildings are at risk of biological corrosion [12]. An additional factor contributing to dampness in the buildings is the geographical location of this part of the former concentration camp. The Vistula River runs two kilometers to the west of the camp, while its tributary, the Pławianka, flows in the south-west. In 2010, the high water in those rivers broke the flood banks and some of the Museum’s buildings were flooded.

Currently, there is no admission to some buildings in sections BI and BII of the former camp due to their poor technical condition. Therefore, intensive conservation work is being conducted to preserve the buildings and to make them again available to visitors.

In order to effectively preserve these historic buildings, it is necessary not only to evaluate their technical condition, but also assess the rate and direction of the biodeterioration process. Due to the fact that the destructive activity of man has been practically eliminated, abiotic environmental factors are solely responsible for deteriorating the buildings. A comprehensive evaluation of the influence of the technical condition of the buildings and atmospheric conditions on biological corrosion in selected brick barracks in the Auschwitz-Birkenau State Museum will make it possible to appropriately manage the protection of these historic buildings.

## Materials and Methods

### Studied buildings

**Figure 1:**
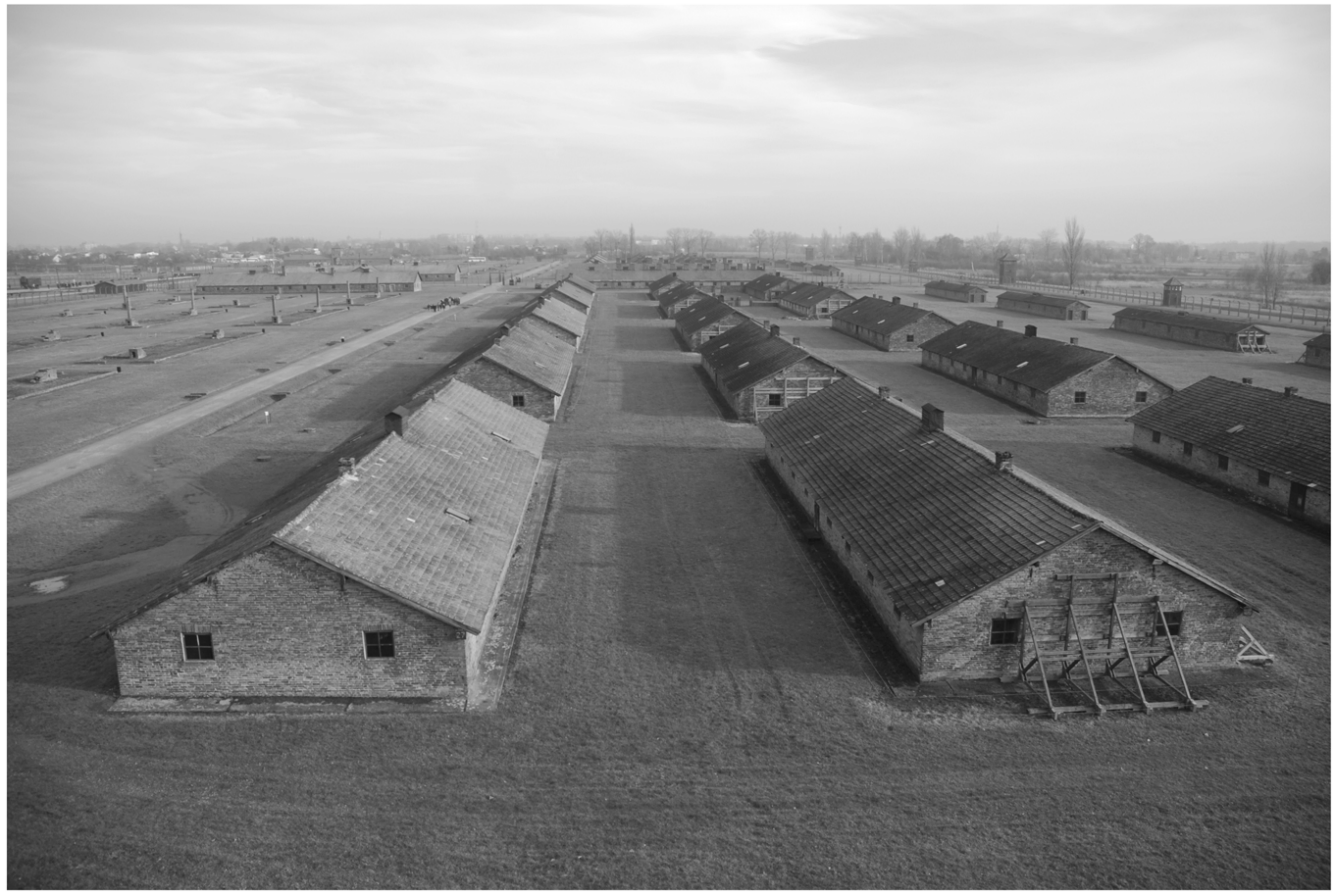
Brick barracks in section BI (photo by Ł. Szoblik).

**Figure 2:**
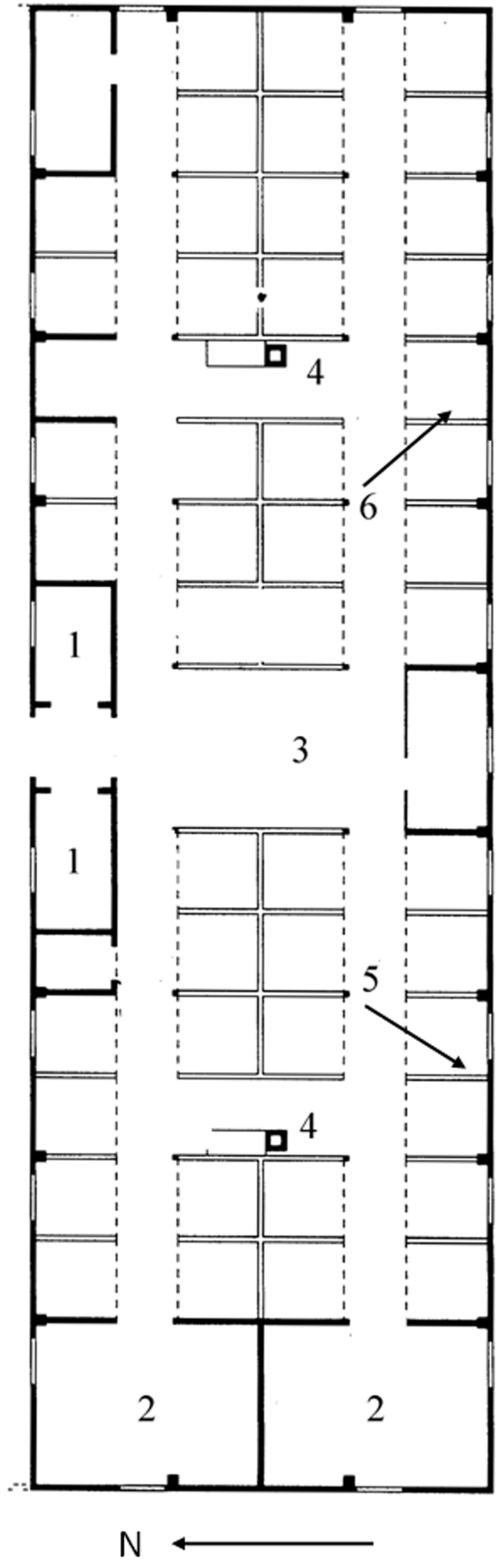
A diagram of the barracks: 1-block master’s room and storeroom; 2-washroom, 3-middle part of building; 4-stove; 5-bunk bed partitions; 6-bunk beds.

The study involved 6 barracks (no. B-70, B-66, B-113, B-114, B-124, and B-138) located in sections BIa and BIb. The studied barracks were selected among all the buildings in this area based on macroscopic assessment of the level of deterioration. The studied buildings were residential brick barracks, with entrances from the north, closed to visitors. The studied area and a diagram of a typical building are presented in Figs. 1 and 2.

Samples for microbiological tests were collected from the interior of the buildings, including both mineral materials (plaster, bricks) and wood (bunk bed planks, door frames, floors). A total of 39 samples were collected. Samples for microbial analysis were taken from the following sites inside buildings: block master’s room and storeroom, washroom, and the main part of building, and included mineral materials such as plaster and brick from construction walls, bunk bed partitions and floors, as well wood from floors and bunk beds (Fig. 2).

Samples for phycological analysis were collected from sites both inside and outside the buildings which were seen to be contaminated with algae. Lichenological and bryological samples were taken from the outside area immediately adjacent to the buildings (concrete strip, rubble, stones), external walls, and the roof.

### Visual evaluation

In each of the studied barracks, visual evaluation was carried out four times from May 2012 to January 2013. Evaluation of the exterior included the condition of concrete strips around the buildings (if present), the condition of moisture insulation (if present), signs of dampness, and the condition of the doors and windows. Inside the barracks, visual evaluation included the condition of the walls, plaster, paint, concrete floor slabs (if present), and wooden elements (bunk beds, doors, floors, roof framing). Particular attention was paid to signs of deterioration such as bulging or crumbling plaster or paint and wood fiber splitting in both structural elements and equipment. Macroscopic examination also involved checking for the presence of biofilms or mold growths, and evaluating the intensity of contamination with bryophytes, lichens, wood-decaying fungi, and algae.

### Deterioration index

Based on the intensity of the observed macroscopic signs of deterioration (bulging or crumbling plaster, wood degradation, and the area affected by fungi, algae, bryophytes, and lichens), a 4-point scale of deterioration was established for the different elements of the buildings: 0 - no changes, 1 - small changes (up to 20% of the examined area), 2 - moderate changes (from 20% to 50% of the area), and 3 - intensive changes (over 50% of the area). Eleven elements were evaluated, both outside (concrete strips, walls, roof framing, and doors) and inside (floors, walls, roof framing) in three different parts of the buildings - the block master’s room and storeroom, the central part of the building, and the washroom. According to equitation (1) the calculation of deterioration index (DI) was proposed:
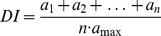
(1)where: n - number of examined elements (n = 11); a_1_, a_2_….a_n_ - score of evaluation for each element (a_max_ = 3).

Examinations were conducted four times: in the spring, summer, autumn, and winter.

### Humidity, moisture, and temperature measurements

Relative humidity (RH) of the air and temperature were measured in five different places inside the buildings, as well as outside the buildings, using a PWT-401 thermohygrometer (Elmetron, Poland).

In selected places at different distances from the ground level, the moisture content of mineral materials was measured. Samples were taken from partition walls in washrooms in barracks B-114 and B-124 from the height of 10 cm, 20 cm, 50 cm, 100 cm, and 160 cm from the ground. Additionally, this parameter was determined for wooden floors in the block master’s room and storeroom. The water content in material samples was determined by the gravimetric method using an RMAC 110/NH moisture analyzer (Radwag, Poland). The results were expressed as percentage content of water by mass.

Each measurement was done in triplicate.

### Microbiological analysis

Microbiological contamination of surfaces inside the barracks was investigated by swabbing 25 cm^2^ areas. Bacterial counts were determined on Tryptic Soy Agar (Merck, Germany) with nystatin, while mold counts on Sabouraud-2% dextrose agar (Merck, Germany) with chloramphenicol. Incubation was conducted at 25±0.5°C for up to 7 days. The results are given as total bacterial and mold counts in CFU 100 cm^−2^ of surface area. The detection limit is 8.0 CFU 100 cm^−2^. In samples for which moisture content was established, microbiological contamination levels were determined by plating the suspension obtained from weighted amounts of the material in the manner described above. The tests were conducted in triplicate and the results are expressed in log CFU g^−1^.

The bacteria were identified by standard methods based on morphological characteristics, Gram staining, and biochemical assays using API tests (bioMerieux, France). Filamentous fungi were identified based on morphological features according to [13], [14]. The taxonomic classification of the predominant microorganisms was confirmed by molecular methods, using the ITS1 (internal transcribed spacer) region sequence for filamentous fungi and the 16S rRNA gene fragment for bacteria [15], [16]. Genomic DNA from isolated bacteria and fungi were extracted using the Genomic Mini Kit and Bead-Beat Micro Gravity (A&A Biotechnology, Gdynia, Poland), respectively, according to manufacturer’s instruction. The amplification of bacterial 16S rRNA gene and were performed with universal primers 27f (5′-AGAGTTTGATCCTGGCTCAG-3′) and 1492r (5′-GGTTACCTTGTTACGACTT-3′). The ITS region were amplified with following primers: ITS1 (5′-TTCGTAGGTGAACCTGCGG-3′) and ITS4 (5′-TCCTCCGCTTATTGATATGC-3′). The PCR reactions were carried out in 50 µl volumes containing 40 pmol of each primer, 1.5 U of RedTaq ReadyMix DNA polymerase (Sigma-Aldrich, St. Louis, MO, USA), 20 ng of template DNA and made up to 50 µl with PCR grade water. The PCR was performed in the MJ Mini Gradient Thermal Cycler (Bio-Rad, Hercules, CA, USA). PCR products were detected by 1% (w/v) agarose gel electrophoresis in 0.5× TBE buffer (Sigma-Aldrich, St. Louis, MO, USA) and purified using Clean Up Mini Kit (A&A Biotechnology, Gdynia, Poland) following the manufacturer purification protocol. The nucleotide sequences of the 16S rRNA genes and ITS regions were obtained using the BigDye Terminator Ready Reaction Cycle Sequencing kit (Applied Biosystems, Foster City, CA, USA). The reaction products were analyzed using an Applied Biosystems model 3730 Genetic Analyzer (Genomed, Warsaw, Poland). The nucleotide sequences were compared with those published on The National Center for Biotechnology Information (NCBI) database and align using the program BLASTN 2.2.27+ (http://www.blast.genome.ad.jp). The 16S rRNA gene and ITS regions sequences of isolated microorganisms were deposited in the GenBank database with the accession numbers: KM 036068 (*Bacillus amyloliquefaciens),* KM 036070 (*B.cereus*), KM 036072 (*B.gibsonii*), KM 036073 (*B.idriensis*), KM 036074 (*B.muralis*), KM 036082 (*Paenibacillus terrigena*), KM 036084 (*Psychrobacillus psychrodurans*), KM 036086 (*Rhodococcus fascians*), KM 036087 (*Sporosarcina aquimarina*), KM 036089 (*Staphylococcus equorum*), KM 036083 (*Pseudomonas fluorescens*), KM 036090, KM 036091, KM036092 (*Engyodontium album*), KM 036093 (*Epicoccum nigrum*).

Wood-decaying fungi were identified *in situ* based on macroscopic growth characteristic according to [2], [17].

### Phycological analysis

Algae were scraped off building surfaces to test tubes with distilled water using soft brushes and fixed with 4% formaldehyde solution. Algae and cyanobacteria groups were identified according to [18], [19], [20], [21], [22].

### Lichenological analysis

Biological material was collected from surfaces outside barracks, and then was examined microscopically. Species identification was based on [23], [24], [25], [26].

### Bryological analysis

Biological material was collected from building structures inside and outside and the morphology of the specimens was evaluated in temporary mounts. The taxa of bryophyte are given based on [27], [28].

### Statistical analysis

Statistical analysis (means, standard deviations) and analysis of the statistical significance of differences (one-way analysis of variance ANOVA) was conducted using ORIGIN v.6.1 (OriginLab Corporation, USA) and Statistica v.10.0 (Stat Soft. Inc., USA) software.

The authors would like to thank the staff of the Auschwitz-Birkenau State Museum for making the facilities available for research.

## Results and Discussion

**Figure 3:**
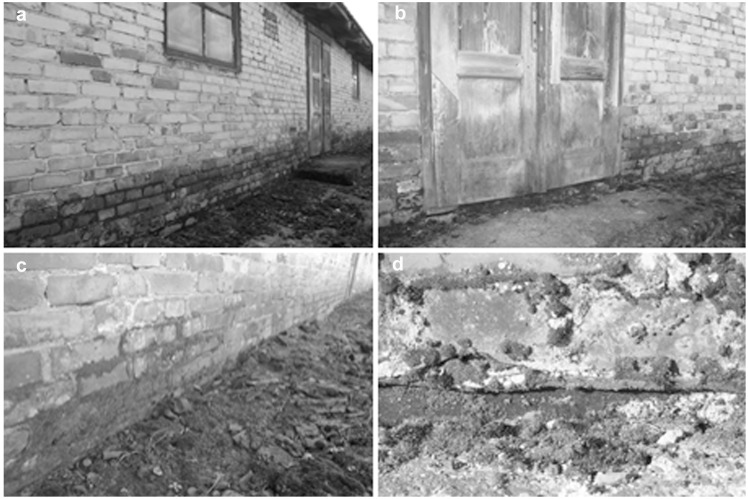
Biodeterioration symptoms of external part of buildings (numerical value of deterioration in a 4-point scale). a) capillary rise of groundwater; area adjacent to the building covered by concrete rubble mixed with soil (3– intensive changes); b) door colonized by algae and cyanobacteria, mosses and lichens on the ground (2– moderate changes); c) concrete rubble mixed with soil, colonization by algae and mosses (3– intensive changes); d) colonization by lichens and mosses, horizontal moisture insulation damaged (3– intensive changes).

The study was conducted from May 2012 to January 2013. Pronounced signs of deterioration were found on the external elements of the barracks, as well as on structural partitions and wooden elements inside them (Table 1). Only two barracks were encompassed by concrete strips, but they were cracked with many holes. The ground surrounding the other barracks was covered by brick and concrete rubble mixed with soil (Fig. 3a, c). In the area immediately adjacent to the buildings, especially on their northern side, large lichen and bryophyte populations were found, which spread onto the walls to a height of approximately 1 m, and also invaded the roofs in isolated colonies (Fig. 3a, d). The area affected by these organisms diminished with distance from the ground. Locally on door and walls the colonization by algae and cyanobacteria were observed (Fig. 3b). Five buildings had some horizontal moisture insulation in the form of tar paper or membrane, but those materials were cracked, especially on the northern side. Despite this insulation, the northern walls of the buildings (B-113, B-114, B-124, and B-138) were damp up to 0.8 m as a result of capillary rise of groundwater (Fig. 3a).

**Table 1:** Technical condition of the buildings and the extent of deterioration.

No.	Exterior of the building							Interior of the building								DI
	Technical condition				Extent of deterioration(on a 4-point scale)				Extent of deterioration (on a 4-point scale)							
	Concretestrip	Horizontalmoistureinsulation	Walldampness	Biofilms	Concretestrip	Wall	Roofframing/door	Biofilms	Block master’sRoom andstoreroom		Central area			Washroom		
									G	W	G	W	P	G	W	
B-66	–	+*	–	ABL	2	2	1	A	1	2	1	3	0	1	1	0.42
B-70	–	+*	–	BL	2	1	0	AF	1	2	1	2	1	2	2	0.42
B-113	–	–	+	BL	3	2	1	AB	3	2	1	2	0	1	2	0.52
B-114	+*	+*	+	BL	3	3	1	AB	3	2	2	2	0	3	2	0.64
B-124	–	+	+	BL	3	2	0	ABF	3	2	3	2	1	3	3	0.67
B-138	+*	+	+	ABL	3	2	2	A	1	2	0	1	0	2	2	0.45

(+) element present; (*) element damaged; A - algae and cyanobacteria; B - bryophytes; L - lichens; F - wood-decaying fungi; G - ground floor; W - wall; P - bunk beds.

Scale: 0- no changes, 1- small changes (up to 20% of the area), 2- moderate changes (from 20% to 50% of the area), 3- intensive changes (over 50% of the area).

**Figure 4:**
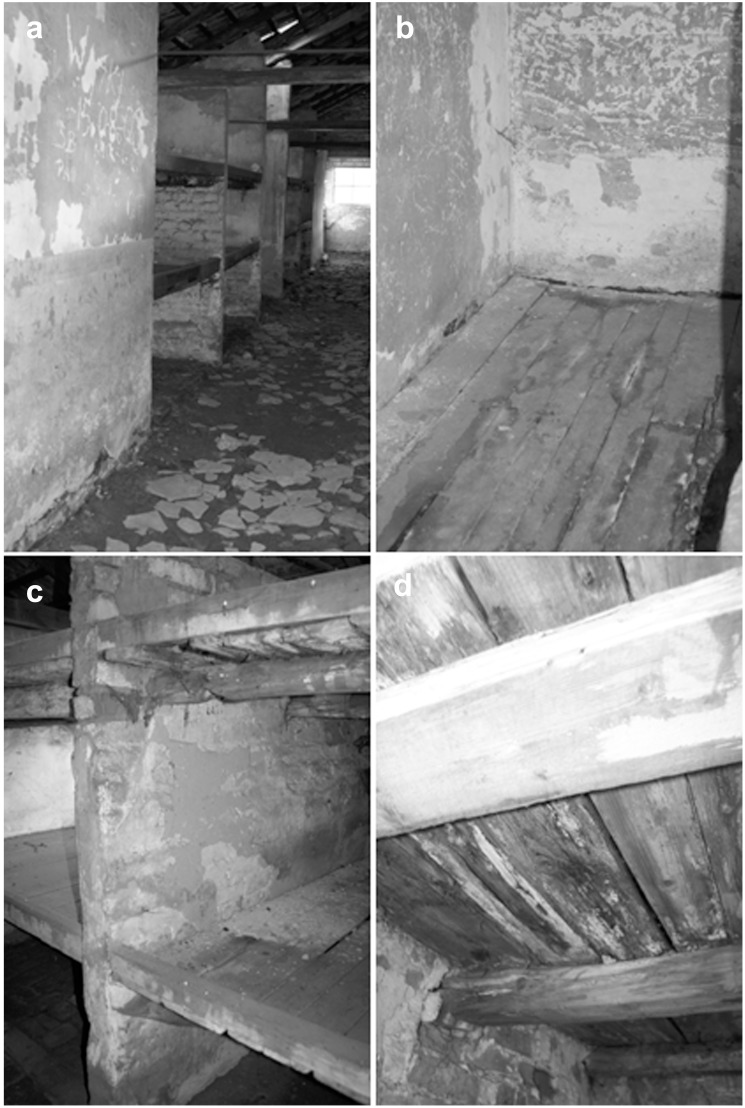
Biodeterioration symptoms of internal part of buildings (numerical value of deterioration in a 4-point scale). a) the middle part of building, damages of the floor (3– ground floor: intensive changes, 2– walls: moderate changes); b) block master’s room and storeroom, damages of the wood floor, algae and cyanobacteria colonization (3– ground floor: intensive changes, 1– walls: small changes); c) bunk bed partitions, bulging and crumbling plaster (3– intensive changes); d) damages of bunk beds board caused by wood-decaying fungi (2– moderate changes, locally).

Inside the brick buildings, bulging and crumbling plaster, peeling paint, crumbling bricks and concrete floor slabs, and wood fiber splitting in bunk beds and floors were observed (Table 1, Fig. 4a–c).). On interior surfaces, no signs of microbial contamination typical of molds or bacteria (biofilms, discolorations) were found. However, the consequences of dampness were visible. Macroscopic effects of wall deterioration were noted in the area immediately adjacent to the floor and up to approximately 0.5 m. Algae populations inside the buildings most often developed on cracked concrete floor slabs, wooden structural elements, and on those bricks which formed the floors in the washrooms (especially near the windows). In building B-70, some wood-decaying fungi were found on wooden bunk beds (Fig. 4a, d).

The highest deterioration index (0.64–0.67), corresponding to intensive corrosion processes, was found for buildings B-114 and B-124. These barracks are located in the western part of the examined area, closest to the Vistula, and are in the worst technical condition. The lowest DI (0.42) was found for buildings B-66 and B-70, located in the eastern part, furthest from the river. The high state of the Vistula River and their tributary Pławianka, especially in Spring, increase groundwater level in areas located close to the rivers. Visual evaluation was conducted four times throughout the study period, but no significant changes in the building condition or biological corrosion extent between the seasons were identified.

**Figure 5:**
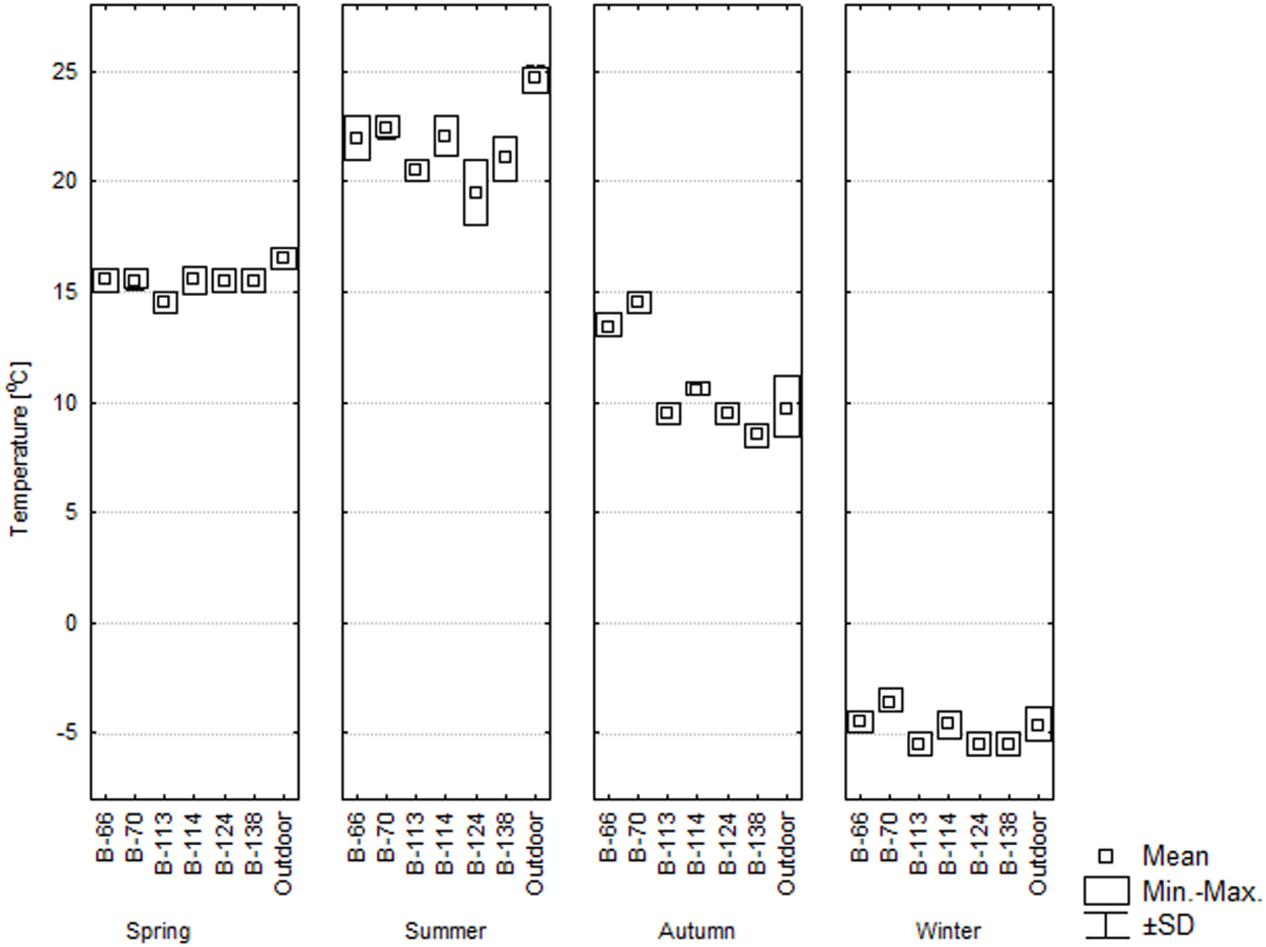
Temperature inside the studied buildings [°C]; SD values did not exceed ±1.4°C.

**Figure 6:**
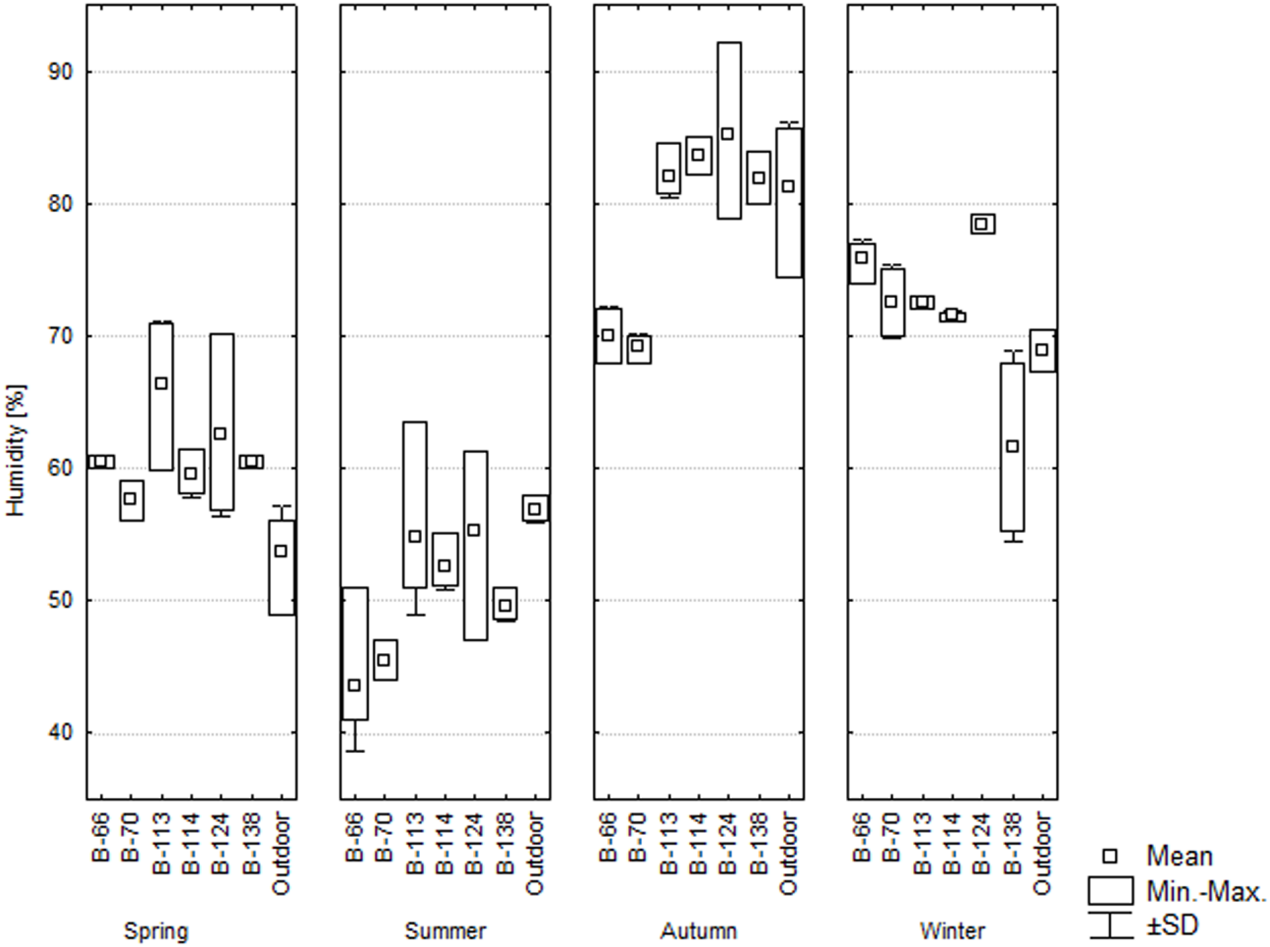
Relative air humidity in the studied buildings [%].

Figs. 5 and 6 present the microclimatic parameters inside and outside the examined buildings. The mean air temperature inside the buildings was from 3.5 to 22.5°C, depending on the season. In the spring, it was similar to the outside temperature. Only in barrack B-113, it was lower by 1.9°C (p<0.05). During the Summer, the temperature inside all the buildings was by 2.5°C-5.4°C lower than outside (p<0.05). In the Autumn, the temperature inside two barracks (B-66 and B-70) was higher by about 5°C than outside (p<0.05), while the temperature in the other buildings was similar to the outside temperature. In the wintertime, temperatures below freezing point were found in all the buildings; in two buildings they were similar to the outside temperature, while in the others they were higher or lower by approximately 2°C (p<0.05). The temperatures inside the buildings were rather stable as variation between different measurement points in a given building did not exceed 3°C.

Throughout the year, relative air humidity in the barracks ranged from 42.8% in the summertime to 85.3% in the autumn. During the spring, RH levels were higher by 3.9%–12.3% inside all the barracks (except for B-70) than outside (p<0.05). In the summertime, RH levels ranged from 43.6% to 54.8% and were lower indoors than outdoors. In the autumn, relative humidity was the highest among the studied seasons, as it exceeded 80% and was similar inside and outside most buildings. Only in barracks B-66 and B-70, RH was considerably lower than outside (by approximately 10%, p<0.05). In the wintertime, relative humidity was higher by approximately 5% inside barracks B-66, B-113, B-114, and B-124 than outdoors (p<0.05). During the spring, summer, and autumn, RH differences between different places inside the barracks reached 14%, which may indicate variation in the dampness of building materials. It was found that locally, especially in the washrooms, relative humidity was very high and exceeded 90% (building B-124 in the autumn). Building B-124 was also characterized by the highest deterioration index (0.67). The humidity and temperature conditions in the studied barracks, especially in the autumn, are conducive to microbial growth [6], [7], [29], [30]. In the wintertime, a relative humidity of more than 70% would also promote the development of microorganisms, but low temperatures considerably inhibit their activity. Such conditions, however, are favorable for psychrotrophs (cold-tolerant organisms).

Due to the fact that the availability of water in the substrate determines the development of microorganisms, moisture content by mass was determined for samples of materials collected at different distances from the ground.

**Figure 7:**
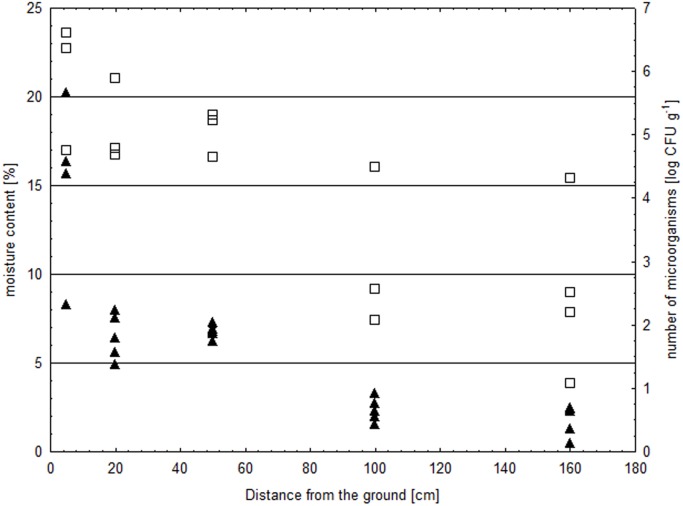
Moisture content and microbial counts in mineral materials depending on distance from the ground: ▴ moisture content [%]; □ number of microorganisms [log CFU g^−1^].

It was found that moisture content by mass decreased with distance from the ground (Fig. 7). Up to a height of 5 cm, moisture content amounted from 8.3% to 20.2%, which indicates that the walls were damp [6]. The brick collected from the washroom of barrack B-124 were characterized by both the highest moisture content (20.2%) and the microbial contamination was 4.0×10^6 ^CFU g^−1^.

The samples of mineral materials collected from areas located 20 cm above ground were also most heavily contaminated with microorganisms (more than 10^6 ^CFU g^−1^ of material). Moisture content in the higher parts of the walls, above 50 cm, did not exceed 3%, which may be considered acceptable [6], while the number of microorganisms was lower by 1.5 to 5 log units than that observed in areas located close to the ground. Moisture content in the wood from the floor of the block master’s room and storeroom in barrack B-124 was from 23.4% to 71%, depending on the sampling site (data not presented). The dampest areas were affected by wood-decaying fungi.

Table 2 presents the results of microbiological examinations, broken down into the various types of materials. In the spring, the surface of mineral materials (bricks, plaster, mortar, paint) was most heavily contaminated in building B-138 (8.4×10^3 ^CFU 100 cm^−2^). Wooden surfaces were characterized by microbial counts of 7.2×10^3 ^CFU 100 cm^−2^. In the summertime, the numbers of microorganisms were higher for most sampled sites, even by 3 log units, which may be linked to increased rainfall before tests. During the autumn, the highest levels of microbiological contamination were found in buildings with the highest DI values (B-113, B-114, and B-124), especially on wooden surfaces. In the wintertime, microbial counts decreased, even by 2 log units, as compared to corresponding samples in the autumn. Generally, the highest microbiological contamination was observed on surfaces with visible signs of deterioration - crumbling plaster and decomposing and crumbling wood. While the recorded counts of microorganisms do not indicate ongoing microbial activity [31], the presence of microorganisms does not exclude the possibility of slow structural changes of these surfaces. Only in the autumn, the total count of microorganisms residing on wood was higher in buildings with signs of deterioration, which were characterized by the highest moisture content in structural elements and relative air humidity. In the other seasons, the tests did not reveal such a pronounced relationship between macroscopic symptoms of deterioration, relative air humidity, and total microbial counts on surfaces.

**Table 2:** Microbial contamination of building surfaces [CFU 100 cm^−2^].

No.		Mineral surfaces				Organic surfaces			
		Spring	Summer	Autumn	Winter	Spring	Summer	Autumn	Winter
B-66	R	8.0–2.4×10^2^	5.8×10^1^–2.4×10^2^	1.7×10^2^–5.4×10^3^	4.9×10^2^–4.6×10^3^	3.7×10^2^–1.2×10^3^	2.4×10^2^–3.5×10^2^	1.6×10^1^–2,2×10^2^	9.6×10^1^–1,7×10^2^
	M	1.4×10^2^	1.7×10^2^	2.8×10^3^	2.8×10^3^	8.9×10^2^	3.0×10^2^	1.3×10^2^	1.2×10^2^
	SD	1.2×10^2^	9.7×10^1^	2.6×10^3^	2.1×10^3^	4.5×10^2^	5.6×10^1^	1.0×10^2^	3.9×10^1^
B-70	R	1.6×10^1^–7.2×10^2^	1.3×10^2^–6,0×10^2^	2.5×10^1^–1.7×10^3^	1.6×10^1^–8.6×10^2^	1.6×10^1^–2.6×10^3^	7.4×10^1^–1.4×10^3^	9.6×10^1^–1,5×10^3^	1.6×10^1^–4,3×10^3^
	M	2.9×10^2^	3.3×10^2^	3.9×10^2^	2.3×10^2^	1.5×10^3^	7.2×10^2^	7.7×10^2^	2.2×10^3^
	SD	2.7×10^2^	1.7×10^2^	6.6×10^2^	3.5×10^2^	1.4×10^3^	9.1×10^2^	9.6×10^2^	3.0×10^3^
B-113	R	1.6×10^1^–2.4×10^2^	2.2×10^1^–1,1×10^3^	1.6×10^1^–1,0×10^3^	1.6×10^1^–7.0×10^1^	1.6×10^1^–2,8×10^3^	1.9×10^2^–1.9×10^3^	2.5×10^1^–4.2×10^3^	1.6×10^1^–6.6×10^2^
	M	6.1×10^1^	2.6×10^2^	2.6×10^2^	3.4×10^1^	9.8×10^2^	7.8×10^2^	1.5×10^3^	2.42×10^2^
	SD	1.0×10^2^	4.5×10^2^	4.2×10^2^	2.3×10^1^	1.6×10^3^	9.4×10^2^	2.4×10^3^	3.7×10^2^
B-114	R	nd	8.4×10^1^-2.4×10^2^	1.6×10^1^–2.4×10^3^	1.6×10^1^–4,1×10^2^	1.6×10^1^–7.2×10^3^	3.0×10^2^–4.6×10^2^	2.5×10^1^–3.9×10^3^	1.6×10^1^–3,9×10^3^
	M	nc	1.7×10^2^	7.6×10^2^	1.6×10^2^	1.4×10^3^	9.5×10^2^	2.1×10^3^	1.3×10^3^
	SD	nc	6.8×10^1^	1.1×10^3^	1.8×10^2^	1.8×10^3^	9.9×10^2^	2.0×10^3^	2.2×10^3^
B-124	R	nd	2.0×10^1^–1.2×10^3^	2.6×10^1^–8,1×10^2^	1.6×10^1^–1,1×10^3^	8.0–1.2×10^2^	2.1×10^1^–8.0×10^2^	5,9×10^2^–2,6×10^3^	3,5×10^2^–9,2×10^2^
	M	nc	3.9×10^2^	2.7×10^2^	2.4×10^2^	6.4×10^1^	3.0×10^2^	1.8×10^3^	6.6×10^2^
	SD	nc	4.7×10^2^	3.2×10^2^	4.8×10^2^	5.6×10^1^	4.4×10^2^	1.1×10^3^	2.9×10^2^
B-138	R	1.6×10^1^–8,4×10^3^	2.2×10^2^–2.9×10^3^	9.6×10^1^–4.3×10^2^	1,1×10^2^–1,7×10^3^	nd	3.1×10^2^–4.4×10^2^	nd	1.3×10^2^–1.5×10^2^
	M	2.4×10^3^	1.0×10^3^	2.5×10^2^	8.8×10^2^	nc	3.7×10^2^	nc	1.4×10^2^
	SD	4.0×10^3^	1.2×10^3^	1.4×10^2^	8.5×10^2^	nc	9.0×10^1^	nc	9.3×10^1^

R - range; M - mean; SD - standard deviation; nd - not detected; nc - not calculated.

The identification of the main microbial species revealed greater biodiversity in inorganic material samples; a total of 70 mold and bacterial species were isolated (20 more than from wood samples). Among the molds, the predominant species belonged to the genera *Cladosporium*, *Alternaria*, *Penicillium*, *Acremonium*, and *Engyodontium*, while the most prevalent bacterial genera were *Bacillus* and *Micrococcus* (Table 3). However, no species were found to be specific to a particular type of material. Some of the isolated bacterial and mold species exhibited very high corrosive potential, e.g., the calcite-forming bacteria *Bacillus muralis*, *B. atrophaeus*, *B. mycoides*, and *B. gibsonii*, and acid-forming molds of the genus *Penicillium*, such as *P.citreonigrum*, *P.corylophilum*, and *P.commune*, which may cause structural changes in materials [32], [33], [34]. Macroscopic evaluation of wooden floors and bunk beds revealed wood-decaying fungi and changes in wood structure such as missing portions of wood, fiber splitting, and cubical cracking (Fig. 4 d). These effects are due to the simultaneous occurrence and succession of different species of wood-decaying fungi (*Poria vaporaria* and fungi of the family *Corticiaceae*). Furthermore, these microorganisms may spread and cause degradation of adjacent elements [35]. Brown rot fungi (*Poria vaporaria*, *Serpula lacrymans*) considerably decrease wood strength (down to 30% of the initial strength after 6 months), which is particularly dangerous in the case of structural elements. In turn, fungi of the family *Corticiaceae* cause superficial wood degradation, and thus they pose a smaller risk to the strength of structural elements [2], [17].

**Table 3:** Predominant microorganisms on inside and outside surfaces of the studied buildings.

Substrate	Type oforganism	Numberof taxa	Predominant taxa
Building interiors			
Mineral^a^	Bryophytes	3	*Tortula muralis,* division *Marchantiophyta*
	Algae andcyanobacteria	35	*Achnanthidium minutissimum, Anomoeoneis vitarea, Apatococcus lobatus, Chlorella vulgaris, Chlorococcum infusionum, Chroococcopsis fluviatilis, Chroococcus varius, C. minor, Cocconeis pseudothumensis, Denticula kuetzingii, Diadesmis contenta, Euastrum sp., Gloeothece palea, Gomphonema parvulum, Leptolyngbya foveolarum, L.notate, Luticola mutica, Neidium ampliatum, Nitzschia debilis, N.vitarea, Nostoc sp., Phormidium aerugineo–caeruleum, P.breve, P.tergestinum, Planothidium lanceolatum, Scytonema drillosiphon, Tolypothrix sp., Trybionella hungarica, Xanthonema sp.*
	Bacteria	19	*Bacillus amyloliquefaciens, B.atrophaeus, B.cereus, B.gibsonii, B.idriensis, B.muralis, B.mycoides, B.pumilus, B.simplex, B.subtilis, Micrococcus luteus, Paenibacillus terrigena, Pseudomonas fluorescens, Psychrobacillus psychrodurans, Rhodococcus coprophilus, Rhodococcus fascians, Sprosarcina aquimarina, Staphylococcus equorum*
	Molds	51	*Acremonium sclerotigenum, A.strictum, A.murorum, Absidia glauca, Alternaria alternata, A.radicina, Aspergillus fumigatus, A.meleus, A.flavus, Botryosphaeria* sp., *Botrytis cinerea, Chaetomium globosum, Curvularia lunata, Cladosporium cladosporiodes, C.herbarum, C.oxysporum, Engyodontium album, Epiccocum nigrum, Fusarium* sp., *Humicola fuscoatra, Penicillium citreonigrum, P.citrinum, P.commune, P.corylophilum, Rhizomucor pusillus, Rhizopus nigricans, Phoma glomerata, Paecilomyces varioti, P.lilacinus, Sordaria funicola, Torula terrestris, Myrodontium keratinophilum, Aureobasidium pullulans*
Organic^b^	Algae andcyanobacteria	11	*Chroococcopsis fluviatilis, Chroococcus minor, Leptolyngbya sp., Achnanthidium minutissimum, Diadesmis contenta, Nitzschia vitrea, Xanthonema montanum, Apatococcus lobatus, Chlorella sp., Coenochloris.*
	Bacteria	12	*Bacillus cereus, B.atrophaeus, B.gibsonii, B.subtilis, B.simplex, B.pumilus, B.mycoides, Micrococcus luteus, Sporosarcina aquimarina, Staphylococcus equorum, S.gallinarum, Paenibacillus terrigena*
	Molds	38	*Acremonium strictum, A.murorum, Alternaria alternata, Aspergillus fumigatus, Chaetomium globosum, Cladosporium cladosporioides, C.herbarum, Engyodontium album, Epicoccum nigrum, Eurotium herbariorum, Humicola fuscoatra, Penicillium citreonigrum, P.citrinum, P.commune, P.fellutanum, Rhizopus nigricans, Sordaria funicola, Trichoderma viride, Ulocladium botrytis, Torula terrestris*
	Wood-decayingfungi	2–4	*Poria vaporaria,* rodzina *Corticiaceae*
**Building exteriors**			
Mineral^a^	Bryophytes	20	*Atrichum undulatum, Barbula unguiculata, Brachytchecium albicans, Bryum argenteum, B.bicolor, B.caespiticium, Calliergonella cuspidate, Cerathodon purpureus, Cirriphyllum piliferum, Dicranella heteromalla, Dryptodon pulvinatus, Encalypta streptocarpa, Eurhynchium hians, Funaria hygrometrica, Marchantia polymorpha, Rhynchostegium murale, Schistidium apoarpum, Tortula muralis*
	Algae andcyanobacteria	9	*Apatococcus lobatus, Chlorella* sp., *Chlorella vulgaris, Chlorococcum infusionum, Chroococcus minor, C.varius, Coenochloris* sp., *Diadesmis contenta, Gloeocapsa sp., Gloeothece* sp.
	Lichens	25	*Acarospora fuscata, Aspicilia contorta subsp. Contorta, Caloplaca citrine, C.decipiens, C.teicholyta, Candelariella aurella, Cladonia cf chlorophaea, C.furcata, Lecanora* cf. *dispersa, Lecanora conizaeoides, Lecidea stigmatea, Lepraria* cf *incana, Lepraria cf. vouauxii, Mycobilimbia tetramera, Peltigera rufescens, Phaeophyscia orbicularis, Physcia caesia, Porpidia soredizodes, Protoblastenia rupestris, Protoparmeliopsis muralis* var. *muralis, Sarcogyne regularis, Strangospora pinicola, Trapelia coarctata, Verrucaria cf nigrescens, V.muralis, V.rupestris*
Organic^b^	Bryophytes	41	*Atrichum undulatum, Barbula convolute, Barbula unguiculata, Brachytchecium albicans, Brachythecium rutabulum, Brachythecium salebrosum,Bryum argenteum, Bryum capillare, Bryum pallescens, Bryum pseudotriquetrum, Calliergonella cuspidate, Ceratodon purpureus, Cirriphyllum piliferum, Climacium dendroides, Dicranella heteromalla, Didymodon fallax, Ditrichum heteromallum, Eurhynchium hians, Fissidens taxifolius, Funaria hygrometrica, Marchantia polymorpha, Plagiomnium cuspidatum, Pseudephemerum nitidum, Rhynchostegium murale, Rhytidiadelphus squarrosus, Schistidium apocarpum, Sciuro-hypnum oedipodium*
	Algae andcyanobacteria	38	*Achnanthidium minutissimum*, *Apatococcus lobatus, Chlorella* sp., *Chlorella vulgaris, Chroococcus minor, Chroococcus varius, Cocconeis pseudothumensis, Diadesmis contenta, Euastrum* sp., *Leptolyngbya* sp., *Nostoc commune, Nostoc microscopicum, Planothidium lanceolatum*
	Lichens	7	*Lecanora conizaeoides, Mycobilimbia tetramera, Protoparmeliopsis muralis* var. *muralis, Strangospora pinicola*

a Bricks, plaster, mortar, roof tiles;

b Wood.

Inside the buildings, also mineral elements (mostly bricks in the washrooms) and wooden floors in the block master’s rooms and storerooms were affected by algae and cyanobacteria. The number of species found on mineral substrates was much higher than those found on wood (35 and 11 taxa, respectively). Irrespective of the type of substrate, the prevalent genera were *Scytonema*, *Apatococcus*, *Chlorella*, *Diadesmis*, and *Chroococcus* (Table 3). Depending on the season, thalli were observed to either shrink or expand on surfaces inside buildings, which was particularly pronounced in the case of *Scytonema drilosiphon*, a cyanobacterium highly sensitive to humidity and temperature fluctuations.

The external parts of the buildings were colonized by bryophytes, lichens, and, to a lesser extent, algae (Fig. 3). They main genera found on mineral surfaces were *Bryum* (bryophytes), *Diadesmis* and *Chlorella* (algae), *Chroococcus* (cyanobacteria) and *Lecanora* and *Protoparmeliopsis* (lichens). A greater diversity of lichen species was found on mineral surfaces, which may result from the considerable variety of the chemical composition of such substrates (mortar, bricks, roof tiles, concrete rubble). Mineral substrates were colonized by 20 bryophyte species, the predominant genera being *Bryum* and *Barbula*, 25 lichen species, mostly of the genera *Lecanora*, *Caloplaca*, and *Lepraria*, as well as 9 species of algae and cyanobacteria. The growth of those microorganisms presents a risk to the durability of the external structural elements of the buildings. As a result of metabolic processes, algae release lactic, oxalic, succinic, acetic, and pyruvic acids, which cause erosion and degradation of the substrates [3], [36]. Biofilms containing algae developing on wooden surfaces lead to wood destruction, as algae accumulate in their cells water periodically available in their environment, thus increasing moisture in the area they cover.

Bryophytes colonizing the surfaces of structural elements damage them mechanically with their rhizoids. Moreover, they maintain elevated moisture of the substrate by accumulating water in their cells, which enables the development of other microorganisms. Lichens considerably accelerate the decomposition of substrates producing, both acidic and alkaline metabolites [37]. As lichens are poikilohydric organisms (capable of absorbing large amounts of water, thus increasing their weight and volume), they break up the surface layer of the substrate. Their rhizoids penetrate the cracks and absorb the chemical elements necessary for growth, thus causing chemical changes in the substrate, sustaining water in pores, and promoting mechanical erosion. Lichen mycobionts produce so-called lichen acids, which may form metal complexes with, e.g., silicates, bind the calcium contained in the substrate, and dissolve SiO_2_ [4].

## Conclusions

Macroscopic evaluation of the studied buildings did not reveal the development of molds or bacteria, but the effects of moisture were noticeable on the walls and floors. Upon visual inspection, considerable deterioration caused by dampness was found in mineral materials, while only local sites of biological corrosion were identified on wood. Biological contamination was present irrespective of the season of the year. Depending on relative air humidity and, to a lesser extent, on temperature, microbial counts varied slightly between the seasons. The degree to which particular elements were affected by algae, bryophytes, and lichens, and the species composition of those organisms were influenced by the type of substrate, availability of sunlight, and moisture content in the surface.

The damages of analysed building were the results of simultaneous action of environmental abiotic factors (water, salt and freeze thaw cycle) and biological factors including algae and cyanobacteria, mosses, lichens, wood-decaying fungi, and in lesser extent by bacteria and moulds. The factors promoting microbial growth inside the buildings were excessive moisture content in the studied surfaces and too high relative air humidity, resulting from ineffective moisture insulation in the buildings. This is confirmed by the higher biological contamination and greater dampness of areas located close to the ground, as well as the higher moisture content in the materials from these areas.

Preventive actions against the development and expansion of microorganisms should first of all include the removal of the technical causes of excessive dampness. To this end, it is advisable to carry out the necessary construction works and regulate the rivers to prevent future flooding of the studied area. However, in all such works, priority should be given to preserving the authenticity of the memorial site.
